# Hangover-Relieving Effect of Ginseng Berry Kombucha Fermented by *Saccharomyces cerevisiae* and *Gluconobacter oxydans* in Ethanol-Treated Cells and Mice Model

**DOI:** 10.3390/antiox12030774

**Published:** 2023-03-22

**Authors:** Eun Jung Choi, Hyeongyeong Kim, Ki-Bae Hong, Hyung Joo Suh, Yejin Ahn

**Affiliations:** 1 Department of Integrated Biomedical and Life Science, Graduate School, Korea University, Seoul 02841, Republic of Koreasuh1960@korea.ac.kr (H.J.S.); 2 Transdisciplinary Major in Learning Health Systems, Department of Healthcare Sciences, Graduate School, Korea University, Seoul 02841, Republic of Korea; 3 Department of Food Science and Nutrition, Jeju National University, Jeju 63243, Republic of Korea

**Keywords:** kombucha, *Saccharomyces cerevisiae*, *Gluconobacter oxydans*, hangover, ethanol, oxidative stress

## Abstract

This study aimed to evaluate the hangover relieving effect of ginseng berry kombucha (GBK) fermented with *Saccharomyces cerevisiae* and *Gluconobacter oxydans* in in vitro and in vivo models. The antioxidant activity and oxidative stress inhibitory effect of GBK were evaluated in ethanol-treated human liver HepG2 cells. In addition, biochemical and behavioral analyses of ethanol treated male ICR mice were performed to confirm the anti-hangover effect of GBK. The radical scavenging activity of GBK was increased by fermentation, and the total ginsenoside content of GBK was 70.24 μg/mL. In HepG2 cells, in which oxidative stress was induced using ethanol, GBK significantly increased the expression of antioxidant enzymes by upregulating the Nrf2/Keap1 pathway. Moreover, GBK (15 and 30 mg/kg) significantly reduced blood ethanol and acetaldehyde concentrations in ethanol-treated mice. GBK significantly increased the levels of alcohol-metabolizing enzymes, including alcohol dehydrogenase and acetaldehyde dehydrogenase. The balance beam test and elevated plus maze test revealed that high-dose GBK significantly ameliorated ethanol-induced behavioral changes. Collectively, GBK exerted a protective effect against ethanol-induced liver damage by regulating the Nrf2/Keap1 pathway.

## 1. Introduction

Alcohol absorbed into the body is metabolized to acetaldehyde and nicotinamide adenine dinucleotide (NADH) by alcohol hydrolase and alcohol dehydrogenase (ADH). Accumulation of acetaldehyde causes hangovers, which include symptoms such as headache, nausea, vomiting and diarrhea, accelerated liver toxicity, liver damage, and fat accumulation. NADH is oxidized to produce the reactive oxygen species (ROS) hydroxyl radical (•OH) and superoxide radical anion (O_2_^•−^), which induce oxidative damage and disrupt the antioxidant system [1]. Excessive production of ROS causes protein and DNA damage, cell death, tissue and organ damage, aging, and various diseases [2]. In addition, ethanol-induced oxidative stress in hepatocytes plays a key role in the development of alcoholic liver disease [3]. Therefore, the Nrf2 signaling pathway, which inhibits ROS generation and prevents lipid accumulation, is attracting attention for the development of drugs for alcoholic liver disease [4]. Acute alcohol intoxication reduces endogenous antioxidants in the liver, and Nrf2 signaling regulates the expression of these antioxidants and reduces oxidative stress [5]. Recently, many people have shown interest in natural plants and drinks that treat hangovers owing to an increase in income and quality of life [6]. Moreover, research is being conducted to identify liver-protective substances derived from natural products that are safe and have excellent antioxidant activity [7,8].

Ginseng fruit that is more than three years old exhibits antioxidant, cardiovascular disease-improving, and antidiabetic effects [9,10,11]. Ginsenosides, polyacetylenes, polysaccharides, proteins, and phenolic compounds are reportedly the main active substances in ginseng and ginseng berries. In particular, ginseng berries are known to have a higher content of ginsenosides, especially Re, than ginseng roots [9,12]. Many glycoside compounds that exist in nature exert a stronger effect when sugar is decomposed and converted into aglycon [13]. Ginsenosides and polyphenols, which are active compounds in ginseng berries, are mainly present as glycosides, and their activity increases when sugar is converted to aglycon, which is when sugar is decomposed or metabolites have fewer sugars [14,15]. It has been reported that the minor ginsenosides Rg2 and Rh1 protect against liver damage by inhibiting inflammation and apoptosis through the activation of the Nrf2 signaling pathway in LPS-stimulated Hepg2 cells [16]. Therefore, we attempted to increase the bioavailability of the active ingredients in ginseng berries by preparing ginseng berry kombucha (GBK) through fermentation by microorganisms. Kombucha is a beverage fermented with a symbiotic culture of bacteria and yeast (SCOBY) by adding sugar to green or black tea extracts [17]. Kombucha is known to promote detoxification and metabolism, and has recently begun to be widely consumed in the United States and Europe [18]. The fermentation metabolites and physiological activity of kombucha depend on the fermentation substrate, microorganisms in the SCOBY, additives, and fermentation method [19]. GBK was fermented by *Saccharomyces cerevisiae* M-5 isolated from candied ginseng and *Gluconobacter oxydans* isolated from commercial kombucha. It has been reported that *S. cerevisiae* M-5, a β-glucosidase-producing strain, is involved in the conversion of ginsenosides and polyphenols, which are the active compounds in ginseng berries. Therefore, GBK fermented with a strain with β-glucosidase activity is expected to have higher antioxidant and hepatoprotective effects than commercially available kombucha.

Previous studies have reported the activity and isolation of a symbiotic community of acetic acid bacteria and osmophilic yeast involved in kombucha production [20]. In addition, a large number of studies have been conducted on physical processing and bioconversion to convert major ginsenosides present in ginseng to minor ginsenosides [21]. This study aimed to develop a functional beverage and analyze its functionality by producing kombucha and converting ginsenosides using two separate strains. The purpose of this study was to investigate the effects of GBK against ethanol-induced liver damage in HepG2 cells and animal models of hangover. In particular, the hepatoprotective and antioxidant effects of GBK in ethanol-treated HepG2 cells were investigated. In addition, behavioral changes, blood ethanol concentration, levels of toxic intermediate metabolites, and activity of alcohol-metabolizing enzymes in a hangover-induced mouse model were analyzed. The study clarifies the biochemical hangover-relieving activity of GBK and may aid in the development of functional hangover-relieving drinks.

## 2. Materials and Methods

### 2.1. GBK Preparation

Ginseng berries were purchased from a ginseng farm in Goesan-gun (Republic of Korea). GBK fermentation was performed according to previous studies [22]. Ginseng berry medium (GBM) containing raw ginseng berries (20 g), sucrose (2 g), ascorbic acid (0.02 g), and 0.54% black tea infusion (20 mL) was sterilized. Sterilized GBM was inoculated with *S. cerevisiae* M-5 and *G. oxydans* at 5% concentration and incubated at 30 °C for 18 d. *S. cerevisiae* M-5 was isolated from sugar-preserved ginseng with high β-glucosidase activity; *G. oxydans* is an acetic acid-producing strain isolated from commercial Kombucha. Both isolated strains are stored in the Nutraceuticals laboratory at Korea University (Seoul, Republic of Korea). The resulting fermented GBK was filtered through a Whatman No. 5 filter and freeze-dried for use in the experiment.

### 2.2. Analysis of Radical Scavenging Activity of Fermented GBK

To measure the antioxidant activity of GBK, 2,2′-azino-bis (3-ethylbenzothiazoline-6-sulfonic acid) (ABTS) and 2,2-diphenyl-1-picrylhydrazyl (DPPH) radical scavenging activities were measured as previously described [23]. The scavenging activity of GBK is expressed as the IC_50_ value, which is the concentration at which radical generation is reduced by 50%.

### 2.3. Analysis of the Content of Ginsenosides in GBK

The ginsenoside content was measured using high-performance liquid chromatography (HPLC, Agilent Technologies, Santa Clara, CA, USA). The conditions used for HPLC analysis were as follows: column, Cadenza CD-C18 (75 × 4.6 mm, 3 μm particle size); UV wavelength, 203 nm; flow rate, 1.2 mL/min; injection volume, 5 μL; and column temperature, 40 °C. For the separation of ginsenosides, 10% and 90% acetonitrile were used as mobile phases with the following gradient conditions: 90–76%:10–24% (0–44 min), 76–60%:24–40% (44–56 min), 60–50%:40–50% (56–79 min), 50–90%:50–10% (79–82 min), and 90%:10% (82–85 min).

### 2.4. Cell Culture

The human liver cell line HepG2 was purchased from the Korea Cell Line Bank (Seoul, Republic of Korea). HepG2 cells were cultured in a CO_2_ incubator (5% CO_2_, 37 °C; Thermo Fisher Scientific, Cleveland, OH, USA) in Dulbecco’s modified Eagle’s medium (Welgene, Seoul, Republic of Korea) supplemented with 10% fetal bovine serum and 1% penicillin/streptomycin.

### 2.5. Cell Viability Assay

To examine the effect of ethanol treatment on cell viability, HepG2 cells were seeded in a 96-well plate at a density of 1 × 10^5^ cells/mL and cultured for 24 h. The cells were treated with ethanol at different concentrations (25, 50, 100, 200, 500, and 750 mM) for 24 h, then the cell viability was measured using a Quanti-Max™ WST-8 Cell Viability Assay kit (BIOMAX, Seoul, Republic of Korea). In addition, to evaluate the protective effect of GBK against ethanol-induced cell damage, the cells were treated with different concentrations of GBK (40, 60, and 80 μg/mL) added with 600 mM ethanol and then subjected to the WST-8 assay.

### 2.6. mRNA Expression Analysis of Genes Related to Oxidative Stress and Alcohol Metabolism

HepG2 cells were seeded into 6-well plates at a density of 1 × 10^5^ cells/mL, cultured for 24 h, then treated with different concentrations of GBK (40, 60, and 80 μg/mL). After treating the cells with GBK added with 600 mM ethanol for 24 h, total RNA was extracted using TRIzol reagent and a quantitative real-time polymerase chain reaction (qRT-PCR) was performed according to a previously described method [24]. The expression of the following genes was analyzed: catalase (CAT, NM_001752.4), superoxide dismutase 1 (SOD-1, NM_000454.4), glutathione peroxidase 1 (Gpx, NM_000581.4), nuclear factor erythroid 2–related factor 2 (Nrf2, NM_001145412.3), kelch-like ECH-associated protein 1 (Keap1, NM_012289.4), alcohol dehydrogenase (ADH, NM_000668.6), aldehyde dehydrogenase (ALDH, NM_000689.5), cytochrome P450 2E1 (CYP2E1, NM_000773.4), and glyceraldehyde-3-phosphate dehydrogenase (GAPDH, NM_001256799.3). GAPDH was used as an endogenous control.

To analyze the expression of proteins related to Nrf2/keap1 signaling, HepG2 cells were lysed using RIPA buffer (Abcam, Cambridge, MA, USA) and the supernatant was collected using centrifugation (10,000× *g*, 10 min, 4 °C). Protein concentration in the supernatant was measured using the BCA Protein Quantification kit (BIOMAX), and western blotting was performed as described previously [25] after adjusting the amount of protein to 40 μg. All antibodies used in the experiment were purchased from Cell Signaling Technology (Beverly, MA, USA) and diluted 1:1000 in 5% skim milk. Antibodies against the following proteins were used: GAPDH (#5174), Nrf2 (#12721), Keap1 (#4678), heme oxygenase-1 (HO-1, #5853), and anti-rabbit IgG, HRP-linked antibody (#7074).

### 2.7. Malondialdehyde (MDA) Assay

HepG2 cells were seeded into 6-well plates at a density of 1 × 10^5^ cells/mL, cultured for 24 h, then treated with different concentrations of GBK (40, 60, and 80 μg/mL). After treating the cells with GBK added with 600 mM ethanol for 24 h, cell supernatant was collected and MDA content was measured using the OxiTec™ TBARS (Lipid Peroxidation) Assay kit (BIOMAX) according to the manufacturer’s instructions.

### 2.8. Animals

Six-week-old (25–30 g) male ICR mice were purchased from Orient Bio (Seongnam, Republic of Korea) and acclimatized for one week in a room with a 12 h light/dark cycle, a temperature of 22 ± 3 °C, and relative humidity of 40–60%. During the adaptation period, a normal diet (Altromin 1310, Altromin, Lage, Germany) was provided along with ad libitum access to sterilized water. Thirty mice were randomly assigned to five groups (n = 6/group): normal group (oral physiological saline administration), control group (ethanol and physiological saline administration), KL group (ethanol and a low concentration (15 mg/kg) of GBK administration), and KH group (ethanol and a high concentration (30 mg/kg) of GBK administration). Mice in all groups, except the normal group, were treated with ethanol. After 30 min of GBK or physiological saline administration, 2 mL/kg of 25% ethanol was orally administered [26]. Blood was collected from the hepatic portal vein 0.5, 1, and 2 h after oral administration of ethanol. Serum was obtained using centrifugation at 2800× *g* and 5 °C. Mice were anesthetized with CO_2_ and dissected to remove liver tissues, which were rapidly frozen, stored at −70 °C, and used for analysis. Animal experiments were approved by the Korea University Institutional Animal Care and Use Committee (KUIACUC-2022-0037).

### 2.9. Analysis of Blood Ethanol and Acetaldehyde Concentrations

After ethanol administration, blood was collected at different times (0.5, 1, and 2 h after administration) and centrifuged at 1800× *g* for 10 min to obtain serum. Then, ethanol and acetaldehyde concentrations were measured using an Ethanol Assay kit (BIOMAX) and Aldehyde Assay kit (Biovision, Mipitas, CA, USA), respectively.

### 2.10. Analysis of ADH and ALDH Activities in the Liver Tissue

ADH and ALDH activities in the liver tissue were determined. Briefly, the liver tissue was washed three times with phosphate-buffered saline, mixed with a volume of 0.1 M Tris-HCl buffer (pH 7.4) 10 times the tissue weight (g) and homogenized with a glass-Teflon grinder under ice cooling. The homogenate was centrifuged at 2600× *g* for 10 min to obtain the supernatant. The ADH and ALDH activities of the supernatant were measured using an Alcohol Dehydrogenase Assay kit (Abcam, Cambridge, UK) and ALDH Activity Assay kit (Abcam), respectively, and the calculation formulas provided in each kit.

### 2.11. Analysis of Serum AST, ALT, Glucose, and LDH Levels

Serum alanine aminotransferase (ALT), aspartate aminotransferase (AST), glucose (Glu), and lactate dehydrogenase (LDH) levels were analyzed using an automatic biochemical analyzer (DRI-CHEM 3500i, Fujifilm, Co., Tokyo, Japan).

### 2.12. Behavioral Analysis Following Ethanol Administration

In the balance beam test (BBT, Figure S1), the beam apparatus consisted of a 1 m wooden beam with a flat surface of 12 mm width and placed on a table at a height of 50 cm using two pillars [27]. A black box was placed at the end of the beam as the finish point, and a 60-watt bulb was installed to illuminate the start point. To adapt the mice to the balance beam, the test was conducted 3 times a day for 5 days. On the day of the experiment, GBK and ethanol were orally administered and the time required for the mice to reach the 80 cm mark was measured. Behavioral analysis was conducted using a timer and video camera.

The elevated plus maze (EPM; Figure S1) test was performed to assess anxiety. The apparatus was made of black acrylic and consisted of two open branches facing each other in the form of a cross and two branches blocked on all sides [28]. This cross maze was installed at a height of approximately 50 cm from the floor, a video camera was placed on the central ceiling to record the animals’ behavior, and the light intensity was adjusted to 20 lx. At the beginning of the experiment, the mice were placed on the open arm of the maze with their heads facing out and allowed to freely explore the maze. Behavior was observed for 5 min, and then the time the mice stayed on the open and closed arms, number of entries and exits from each arm, and total moving distance were measured using the EthoVision program (Noldus Information Technology, Wageningen, The Netherlands).

### 2.13. Statistical Analysis

Experimental data of the in vitro tests are represented as mean and standard deviation, and data of the in vivo tests are represented as mean and standard error of the mean (SEM). Statistical significance between the groups and the average were analyzed using one-way analysis of variance (ANOVA) followed by Tukey’s multiple test using SPSS (version 10.0; SPSS Inc., Chicago, IL, USA). The level of significance was set at *p* < 0.05.

## 3. Results

### 3.1. Fermentation-Mediated Changes in Radical Scavenging Activity and Ginsenoside Content

**Figure 1:**
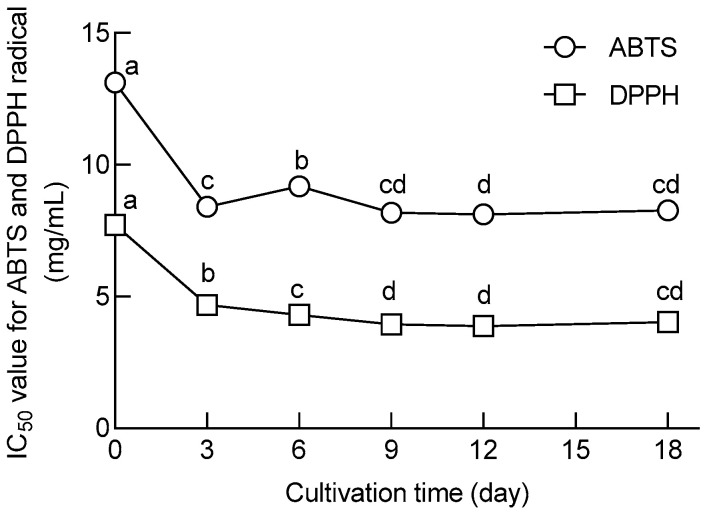
IC_50_ values for ABTS and DPPH radicals during the fermentation of ginseng berry kombucha (GBK). Values are mean ± standard deviation (SD) of each group. Different letters indicate a significant difference at *p* < 0.05 (Tukey’s test). ABTS, 2,2′-azino-bis (3-ethylbenzothiazoline-6-sulfonic acid); DPPH, 2,2-diphenyl-1-picrylhydrazyl.

During GBK fermentation, IC_50_ values for ABTS and DPPH radical scavenging tended to decrease as fermentation progressed (Figure 1). On day 12 of fermentation, the IC_50_ values for ABTS (8.12 mg/mL) and DPPH (3.89 mg/mL) radical scavenging were the lowest (*p* < 0.05) than before fermentation.

As shown in Table 1, the total ginsenoside content of GBK was 70.24 μg/mL. The ginsenosides found in GBK were Rh1, Rg2 (Rg2s and Rg2r), Rg3 (Rg3s and Rg3r), and Re at concentrations 16.81, 12.63, 9.10, and 8.03 μg/mL, respectively. It seems that ginsenosides contained in GBK are the primary substances that remove radicals.

### 3.2. Effect of GBK on the Viability of Ethanol-Treated HepG2 Cells

**Figure 2:**
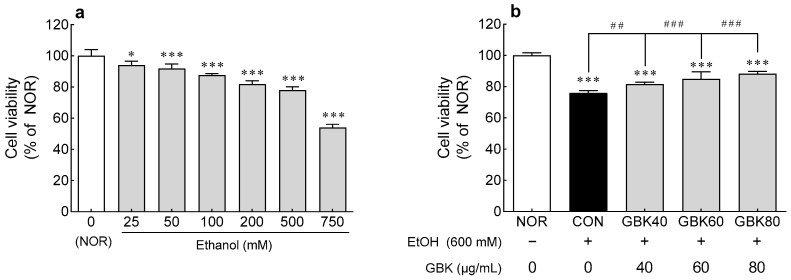
Effects of (**a**) ethanol alone and (**b**) GBK and ethanol mixed treatment on the viability of HepG2 cells. HepG2 cells were treated with GBK in the presence or absence of ethanol for 24 h. Values are mean ± SD. * *p* < 0.05 and *** *p* < 0.001 vs. normal group; ^##^ *p* < 0.01 and ^###^ *p* < 0.001 vs. control group (ANOVA followed by Tukey’s test). EtOH, ethanol.

Figure 2a shows the survival rate of HepG2 cells after ethanol treatment. Ethanol treatment decreased HepG2 cell viability in a concentration-dependent manner. In particular, when the ethanol treatment concentration was 600 mM or more, the cell viability was less than 80% compared to the NOR group (*p* < 0.001). Therefore, cells were treated with 600 mM ethanol to induce oxidative stress.

As a result of measuring the effect of GBK on ethanol-induced hepatocellular damage (Figure 2b), the survival rate was found to be significantly increased by GBK in a concentration-dependent manner when compared to that of control cells treated only with ethanol (*p* < 0.001). Treatment with 80 μg/mL GBK showed cell viability of approximately 88%. Taken together, GBK exhibited hepatoprotective effects against ethanol-induced cell damage.3.3. Effects of GBK on Oxidative Stress Elimination and Alcohol Metabolism-Related Gene Expression in Ethanol-Treated HepG2 Cells.

**Figure 3:**
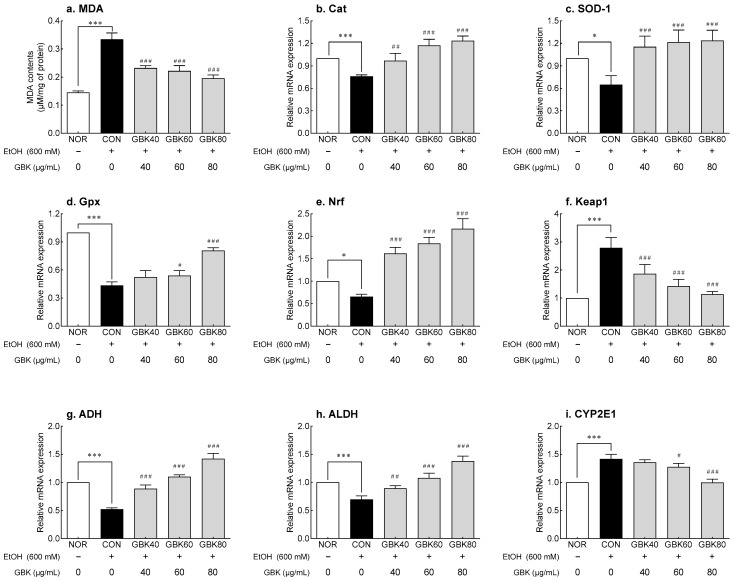
Effects of GBK on (**a**) malondialdehyde content and mRNA expression of factors related to (**b**–**f**) oxidative stress and (**g**–**i**) alcohol metabolism in ethanol-treated HepG2 cells. HepG2 cells were treated with GBK in the presence or absence of ethanol for 24 h. Values are mean ± SD. * *p* < 0.05 and *** *p* < 0.001 vs. NOR group; ^#^ *p* < 0.05, ^##^ *p* < 0.01, and ^###^ *p* < 0.001 vs. CON group (ANOVA followed by Tukey’s test). EtOH, ethanol; MDA, malondialdehyde; Cat, catalase; SOD-1, superoxide dismutase-1; Gpx, glutathione peroxidase; Nrf2, nuclear factor-erythroid 2 related factor 2; Keap1, Kelch-like ECH-associated protein 1; ADH, alcohol dehydrogenase; ALDH, aldehyde dehydrogenase; CYP2E1, Cytochrome P450 2E1.

Treatment with 600 mM of ethanol significantly increased the production of MDA, an intracellular lipid oxidation product, in HepG2 cells (*p* < 0.001). On the contrary, treatment with GBK significantly decreased the production of MDA in a concentration-dependent manner (*p* < 0.001; Figure 3a). The expression of antioxidant enzymes related to the removal of oxidation products and ROS was significantly increased by ethanol treatment when compared to normal cells (*p* < 0.05 and *p* < 0.001, respectively; Figure 3b–d). GBK treatment significantly increased the expression of Cat (*p* < 0.01 and *p* < 0.001, respectively), Sod-1 (*p* < 0.001), and Gpx (*p* < 0.05 and *p* < 0.001, respectively) when compared to control cells in a dose-dependent manner. The ethanol-induced increase in MDA production seemed to be suppressed by GBK-induced increase in CAT, SOD-1, and GPx expression. In addition, we analyzed the expression of genes related to the Nrf2/Keap1 signaling pathway, which is the main signaling pathway that regulates the intracellular oxidation reaction (Figure 3e,f). The expression of the Nrf2 and keap1 genes in the GBK (60, and 80 μg/mL)-treated group was significantly different from that in the control cells (*p* < 0.05 and *p* < 0.001, respectively).

As a result of examining the mRNA expression level of factors related to alcohol metabolism, the expression of ADH and ALDH increased significantly when compared to normal cells due to ethanol treatment (*p* < 0.001; Figure 3g,h). On the other hand, GBK significantly increased the expression of ADH and ALDH in a dose-dependent manner when compared to control cells (*p* < 0.01 and *p* < 0.001, respectively). In addition, ethanol treatment significantly increased the expression of CYP2E1 when compared to normal cells (*p* < 0.001; Figure 3i). GBK (60, and 80 μg/mL) treatment group significantly decreased CYP2E1 expression when compared to control cells (*p* < 0.05 and *p* < 0.001, respectively).

### 3.3. Effects of GBK on Oxidative Stress Elimination-Related Protein Expression in Ethanol-Treated HepG2 Cells

**Figure 4:**
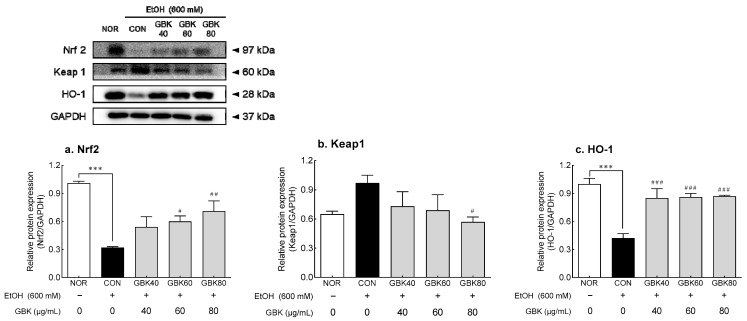
Effect of GBK on the protein expression of (**a**) Nrf2, (**b**) Keap1, and (**c**) HO-1 in ethanol-treated HepG2 cells. HepG2 cells were treated with GBK in the presence or absence of ethanol for 24 h. Values are mean ± SD. *** *p* < 0.001 vs. NOR group; ^#^ *p* < 0.05, ^##^ *p* < 0.01, and ^###^ *p* < 0.001 vs. CON group (ANOVA followed by Tukey’s test). EtOH, ethanol; Nrf2, nuclear factor-erythroid 2 related factor 2; Keap1, Kelch-like ECH-associated protein 1; HO-1, heme oxygenase-1.

GBK treatment increased the expression of Nrf2 (*p* < 0.001) and decreased the expression of Keap1 (*p* < 0.001) in a concentration-dependent manner when compared to the control group (Figure 4). Moreover, the protein expression of Nrf2 and HO-1, which was decreased in the ethanol-treated group, was significantly increased in HepG2 cells by GBK treatment (*p* < 0.01, *p* < 0.05, and *p* < 0.001, respectively). However, GBK (80 μg/mL) treatment significantly reduced Keap1 protein expression, which was increased by ethanol treatment (*p* < 0.05). Collectively, GBK exhibited a protective effect against liver damage caused by ethanol-induced oxidative stress by increasing the expression of antioxidant enzymes through the activation of the Nrf2/Keap1 signaling pathway.

### 3.4. Effects of GBK on Ethanol and Acetaldehyde Concentrations in the Blood of Ethanol-Treated Mice

**Figure 5:**
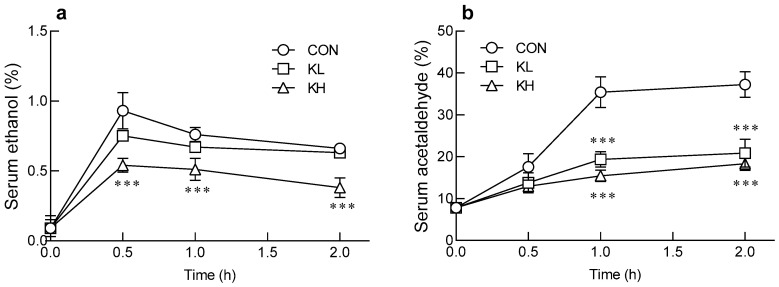
Effect of GBK on serum (**a**) ethanol and (**b**) acetaldehyde levels in mice. Values are mean ± standard of the mean (SEM). GBK was administered orally 30 min before ethanol administration. *** *p* < 0.001 vs. CON group (ANOVA followed by Tukey’s test). CON, control group; KL, low-dose GBK group (15 mg/kg); KH, high-dose GBK group (30 mg/kg).

GBK was administered at low (KL, 15 mg/kg) and high (KH, 30 mg/kg) doses, and after 30 min, 25% ethanol was orally administered. After 0.5, 1, and 2 h, blood ethanol and acetaldehyde concentrations were measured (Figure 5). Compared to the normal group, blood ethanol concentration (%) rapidly increased until 30 min after ethanol administration and then slowly decreased, whereas the concentration of acetaldehyde (%), an alcohol oxidation product, increased until 30 min after ethanol administration and remained high.

Oral administration of GBK suppressed ethanol-induced increase in blood ethanol and acetaldehyde concentrations. High-dose GBK administration (KH) significantly lowered blood ethanol concentration when compared to the control group (*p* < 0.001). After 1 h, the concentration of acetaldehyde was significantly lower in the KH group than in the control group (*p* < 0.001). The administration of GBK not only suppressed the increase in blood ethanol concentration, but also suppressed the increase in the concentration of acetaldehyde, the causative agent of hangover.

### 3.5. Effects of GBK on ADH and ALDH Activities in the Liver of Ethanol-Treated Mice

**Figure 6:**
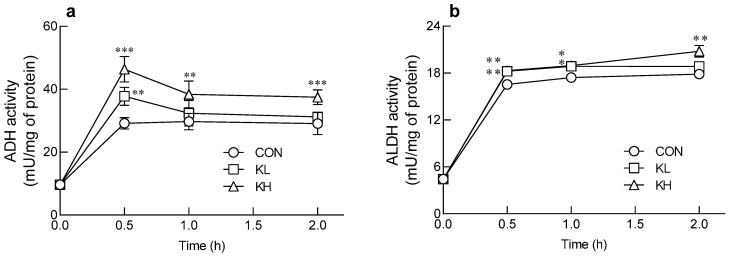
Effect of GBK on (**a**) ADH activity and (**b**) ALDH activity in mouse liver tissues. Values are mean ± SEM. GBK was administered orally 30 min before ethanol administration. * *p* < 0.05, ** *p* < 0.01, and *** *p* < 0.001 vs. CON group (ANOVA followed by Tukey’s test). ADH, alcohol dehydrogenase; ALDH, acetaldehyde dehydrogenase; CON, control group; KL, low-dose GBK group (15 mg/kg); KH, high-dose GBK group (30 mg/kg).

Figure 6 shows changes in ADH and ALDH enzyme activities in mouse livers after GBK administration. ADH is an enzyme that is primarily involved in alcohol metabolism and converts alcohol into acetaldehyde. The activity of ADH in the liver tissue increased until 30 min after ethanol administration but decreased thereafter (Figure 6a). Similarly, the activity of ALDH, which is involved in the decomposition of acetaldehyde, the hangover causative metabolite, rapidly increased until 30 min after ethanol administration and showed little change until 2 h (Figure 6b).

The KH group showed significantly higher ADH (*p* < 0.01 and *p* < 0.001, respectively) and ALDH (*p* < 0.05 and *p* < 0.01, respectively) enzymatic activities than the control group. On the other hand, ADH activity was significantly higher in the KL group than in the control group until 30 min after ethanol administration (*p* < 0.01) but decreased thereafter to the enzyme activity similar to that in the control group. Similarly, ALDH activity was significantly higher in the KL group than in the control group until 30 min and 1 h after ethanol administration (*p* < 0.01 and *p* < 0.001, respectively). GBK (30 mg/kg) increased the activity of the enzymes involved in the degradation of alcohol and acetaldehyde.

### 3.6. Effects of GBK on AST, ALT, and LDH Levels in the Blood of Ethanol-Treated Mice

Table S1 shows GBK-induced changes in AST, ALT, glucose, and LDH levels in the blood of ethanol-treated mice. Serum levels of AST and ALT, which are used as indicators of liver damage, rapidly increased until 30 min after ethanol administration, but decreased thereafter. Contrarily, AST level after 30 min of ethanol administration was significantly lower in both the KL and KH groups than in the control group (*p* < 0.001). Serum ALT levels were significantly decreased (*p* < 0.01) only in the KH group. However, no significant differences were observed in AST, ALT, glucose, and LDH levels 1 and 2 h after ethanol administration (Table S1). The reduction in ALT and AST levels by oral GBK administration demonstrates an inhibitory effect of GBK on ethanol-induced liver damage.

### 3.7. Effects of GBK on Ethanol-Induced Behavior

**Figure 7:**
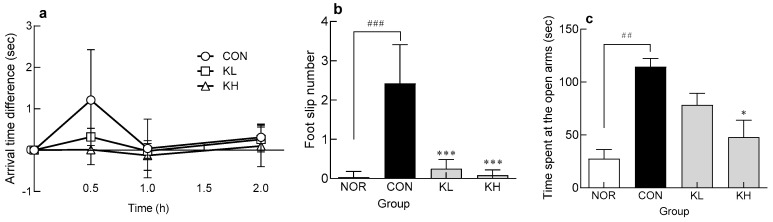
Effect of GBK on ethanol-induced behavioral changes in mice. (**a**,**b**) The balance beam test and (**c**) elevated plus maze test. Values are mean ± SEM. GBK was administered orally 30 min before ethanol administration. ^##^ *p* < 0.01 and ^###^ *p* < 0.001 vs. NOR group; * *p* < 0.05 and *** *p* < 0.001 vs. CON group (ANOVA followed by Tukey’s test). CON, control group; KL, low-dose GBK group (15 mg/kg); KH, high-dose GBK group (30 mg/kg).

The behavior of mice after ethanol administration was analyzed using BBT and EPM tests. Using BBT, the difference in the time taken to reach the 80 cm mark on the beam before and after ethanol administration was measured (Figure 7a). The difference in time to reach the 80 cm mark 30 min after ethanol administration increased by 1.2 s in the control group but increased by 0.32 s and 0.01 s when administered with KL and KH, respectively. Additionally, the number of times the foot slipped before reaching the destination was counted 30 min after ethanol administration (Figure 7b). Ethanol administration significantly increased the number of foot slips (*p* < 0.001), but it was significantly decreased compared to the control group due to GBK (15 and 30 mg/kg) administration (*p* < 0.001).

The EPM test was used to measure the time spent in open and confined spaces to analyze the behavior of mice in terms of exploring new environments and avoiding bright light and open spaces. After 30 min of ethanol administration, control group mice spent significantly more time at the open arms (*p* < 0.01) compared to normal group mice (Figure 7c). When GBK was administered, the time spent at open arms was reduced in a concentration-dependent manner compared to the control group. In particular, KH group mice spent more than twice as much time in the open arms as control group mice (*p* < 0.05). Administration of GBK (30 mg/kg) minimized the behavioral changes caused by ethanol consumption.

## 4. Discussion

In the body, alcohol metabolism is regulated by enzymes, such as ADH, and a microsomal ethanol oxidation system, and generates free radicals that affect the antioxidant system [29]. In the liver tissue, alcohol-induced reduction in antioxidants leads to liver damage by an increase in lipid peroxidation and protein oxidative damage. Additionally, alcohol consumption induces the production of ROS during CYP2E1 pathway oxidation [30]. Cells reduce the production of ROS and suppress the accumulation of lipid peroxides in the body through enzymatic oxidative defense mechanisms, which include SOD, CAT, and Gpx. The expression of these antioxidant enzymes is regulated by the Nrf2/Keap1 signaling pathway; through the activation of Nrf2, the expression of antioxidant enzymes is increased to suppress oxidative stress [31,32]. It has been reported that ginsenoside Rg1 prevents CCl4-induced acute liver damage by inhibiting oxidative stress and inflammatory responses through activation of the Nrf2 signaling pathway [33]. In this study, we found that GBK treatment effectively inhibited ethanol-induced oxidative stress by increasing the expression of antioxidant enzymes via the upregulation of the Nrf2/keap1 pathway.

Alcohol is converted to acetaldehyde by ADH in the body and then oxidized to acetic acid by ALDH, which is excreted as CO_2_ and in urine [34]. It is known that toxicity is caused not only by alcohol but also by acetaldehyde produced during alcohol metabolism [35]. Acetaldehyde is a highly toxic and reactive substance that causes alcohol-induced liver damage and is a major cause of hangovers [36]. AST and ALT activities are increased in damaged hepatocytes, and they are released from the liver into the blood. During alcohol-induced toxicity, AST and ALT are released and result in severe damage to liver tissue membranes [37]. Lee at al. [38] reported that ingestion of black red ginseng mixture in ethanol-administered SD-rats increased the activities of ADH and ALDH in the liver, thereby lowering the concentration of ethanol and acetaldehyde in the blood. In addition, in clinical studies, consumption of red ginseng drink lowered blood alcohol concentration 30 min after drinking and improved hangover symptoms [39]. Here, GBK was involved in relieving hangover by promoting ADH and ALDH activities in the liver and effectively reducing blood alcohol and acetaldehyde concentrations. During this process of ethanol metabolism, CYP2E1, a cytochrome enzyme, is activated and an oxidation reaction through CYP2E1 promotes excessive ROS production, resulting in liver damage [40]. Previous studies have reported an increase in oxidative stress by overexpression of CYP2E1 by ethanol treatment in Hepg2 cells [41]. Lee et al. [42] reported that administration of lactic acid bacteria in Hepg2 cells suppressed oxidative damage in the liver by inhibiting lipid peroxidation by CYP2E1 overexpressed with ethanol. Similarly, GBK reduced alcohol-induced oxidative stress by suppressing the expression of CYP2E1 and increasing the expression of antioxidant enzymes in Hepg2 cells (Figure 3).

Alcohol consumption affects the central nervous system and causes physiological and behavioral changes. Alcohol suppresses the functions of the central nervous system, including the suppression of excitatory neurons and inhibitory neurons [43]. Depending on the person, particularly differences in sensitivity to alcohol, excitatory behaviors are induced by the suppression of inhibitory neurons and sedative behaviors are induced by the suppression of excitatory neurons [44]. During the hangover state, decreased activity, temperature fluctuations, anxiety-like behaviors, and pain perception disorders appear [34]. Zhao et al. [45] reported that administration of Korean red ginseng extract improved ethanol-induced anxiety-related behaviors in SD-rats and inhibited the secretion of stress hormones such as corticosterone. In the present study, GBK administration minimized behavioral changes, as analyzed using BBT and EPM test 30 min after ethanol administration. GBK effectively decreased ethanol-induced behavioral changes by reducing hangover-causing metabolites through its antioxidant action.

The hangover-relieving effect of GBK appeared to be due to an increase in the content of polyphenols and ginsenosides during fermentation. Previous studies reported that polyphenol content in natural products, such as black tea, green tea, and ginseng berries, used in kombucha production is increased by the fermentation process, resulting in increased antioxidant activity [46,47]. Similarly, we found that fermentation increased the radical scavenging activity of GBK during fermentation (Figure 1). As the content of phenolic substances in the extract increases, the radical scavenging activity of ABTS and DPPH increases, and the radical scavenging activity is affected by the content of hydrophilic or hydrophobic substances contained in the sample [48]. In this study, GBK showed a higher DPPH radical scavenging activity than ABTS radical, but the trend according to fermentation time was similar. This difference in radical scavenging activity seems to be due to the various contents of ginsenoside contained in GBK. Our previous study confirmed that fermentation of ginseng berries by *S. cerevisiae* M-5 increased total polyphenol content, especially gallic acid, 3,4-dihydroxybenzoic acid, and chlorogenic acid. Chlorogenic acid and 3,4-dihydroxybenzoic acid, whose contents were increased during GBK fermentation, have been previously reported as antioxidants [49], and ginsenosides are also reportedly involved in the increased antioxidant activity [50]. In this study, the content of minor ginsenosides in GBK was also increased by the β-glucosidase activity of *S. cerevisiae* M-5 strain. β-Glucosidase cleaves the site at which the sugar chain is linked to the glycoside ginsenoside of red ginseng and converts it to the non-glycoside ginsenoside, thereby improving the absorption rate of ginsenoside [51]. GBK contains large amounts of Rh1 and Rg2 (Table 1). Rh1 and Rg2 are minor ginsenosides that can be converted from the major ginsenosides Re and Rg1, and biological conversion of ginsenosides appears to have occurred during GBK fermentation. Park et al. [52] demonstrated that Rg3 and Rh2 ginsenosides protected against alcohol-induced oxidative damage through the regulation of the MAPK pathway in mouse TIB-73 hepatocytes. In addition, it was reported that the ginsenoside Rg1 inhibits alcohol-induced liver fibrosis through the expression of antioxidant enzymes via the activation of the Nrf2 pathway [53]. The ginsenoside contained in GBK helps to remove radicals generated by alcohol, and it seems to have an effect on hangovers.

## 5. Conclusions

GBK effectively inhibited ethanol-induced oxidative stress and protected against hepatocyte damage through the upregulation of the Nrf2/keap1 signaling pathway. In addition, GBK administration suppressed ethanol-induced behavioral changes and regulated the activity of enzymes involved in alcohol metabolism to relieve hangover. Thus, GBK can be used as a hangover relieving drink. Moreover, it is necessary to verify the efficacy of GBK through clinical trials as well as additional studies to identify the interaction between the activity of enzymes involved in alcohol metabolism and the expected active ingredient of GBK using single ginsenoside compounds.

## Figures and Tables

**Table 1:** Content (µg/mL) of ginsenosides of ginseng berry kombucha (GBK).

Ginsenoside	Rg1	Re	Rf	Rg2(s)	Rg2(r)	Rg3(s)	Rg3(r)	Rg5	Rg6
Contents (µg/mL)	1.26 ± 0.03	8.03 ± 0.10	0.45 ± 0.02	8.15 ± 0.07	4.48 ± 0.15	7.34 ± 0.15	1.76 ± 0.07	3.30 ± 0.22	1.84 ± 0.07
**Ginsenoside**	**Rb1**	**Rc**	**Rd**	**Rh1(s)**	**Rh4**	**Rk1**	**F2**	**CK**	**Total**
Contents (µg/mL)	0.96 ± 0.06	1.26 ± 0.11	1.98 ± 0.28	16.81 ± 0.88	4.26 ± 0.29	1.69 ± 0.07	5.87 ± 0.05	0.88 ± 0.37	70.24 ± 2.14

Values are mean ± standard deviation of each group. CK: compound K.

## Data Availability

Data is contained within the article or Supplementary Material.

## Supplementary Materials

The following supporting information can be downloaded at: https://www.mdpi.com/article/10.3390/antiox12030774/s1, Figure S1: Schematics illustrating the apparatus of the balance beam test (left) and elevated plus maze test (right); Table S1: Effect of ginseng berry kombucha on serum AST, ALT, GLU, and LDH levels in mice.
