# Supplementary material for: Hangover-Relieving Effect of Ginseng Berry Kombucha Fermented by Saccharomyces cerevisiae and Gluconobacter oxydans in Ethanol-Treated Cells and Mice Model
Source: Antioxidants (Basel). 2023 Mar 22;12(3):774. doi: 10.3390/antiox12030774 (PMC10045427; doi:10.3390/antiox12030774)
Supplement: Supplementary file 1 [file antioxidants-12-00774-s001.zip › antioxidants-2225695-supplementary.pdf]

Supplementary data

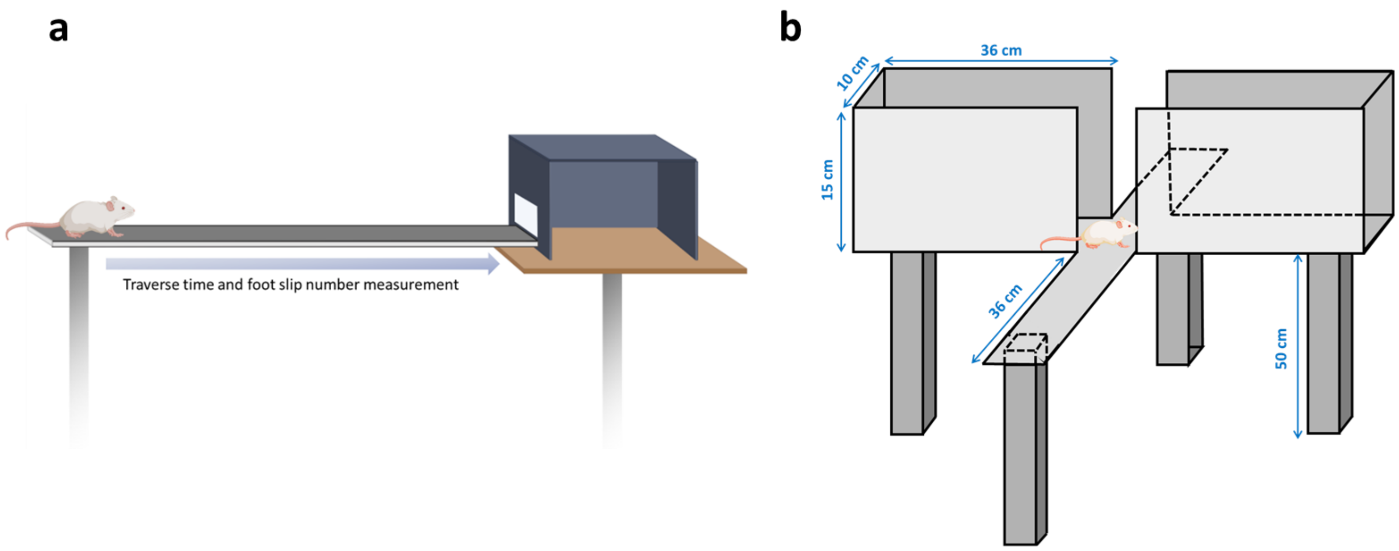

Figure S1. Schematics illustrating the apparatus of (a) the balance beam test and (b) elevated plus maze test.

Table S1. Effect of ginseng berry kombucha on serum AST, ALT, GLU, and LDH levels in mice

Time (h)		0	0.5	1	2
AST (U/L)	CON	64.67 ± 14.24	460.00 ± 4.04	110.67 ± 54.78	68.00 ± 8.50
	KL		112.33 ± 29.91***	65.33 ± 2.02	69.50 ± 9.20
	KH		76.50 ± 2.25***	74.25 ± 3.75	80.00±2.65
ALT (U/L)	CON	33.00 ± 10.21	123.67 ± 18.91	49.67 ± 29.28	45.00 ± 14.64
	KL		64.67 ± 2.90	34.50 ± 7.84	29.67 ± 5.01
	KH		44.33 ± 14.92**	41.33 ± 4.86	44.00 ± 5.20
GLU (U/L)	CON	163.67 ± 32.83	236.00 ± 26.35	227.33 ± 38.02	144.00 ± 7.81
	KL		141.00 ± 9.85	127.00 ± 5.50	159.33 ± 5.35
	KH		189.67 ± 12.06	228.67 ± 10.07	150.00 ± 20.22
LDH (U/L)	CON	913.00 ± 46.95	881.50 ± 114.72	654.00 ± 115.94	628.33 ± 108.95
	KL		1175.00 ± 130.81	1011.67 ± 300.31	757.00 ± 105.93
	KH		1053.00 ± 267.79	1370.25 ± 264.96	1523.50 ± 78.36

Values are mean ± standard error of the mean. GBK was administered orally 30 min before ethanol administration. ***$p<0.001$ vs. CON group (ANOVA followed by Tukey's test). ALT, alanine aminotransferase; AST, aspartate aminotransferase; GLU, glucose; LDH, lactate dehydrogenase; CON, control group; KL, low-dose GBK group (15 mg/kg); KH, high-dose GBK group (30 mg/kg).
